# Experimental Analysis of the Mechanism of Hearing under Water

**DOI:** 10.1155/2015/526708

**Published:** 2015-12-06

**Authors:** Shai Chordekar, Liat Kishon-Rabin, Leonid Kriksunov, Cahtia Adelman, Haim Sohmer

**Affiliations:** ^1^Department of Communication Disorders, Sackler Faculty of Medicine, Tel Aviv University, The Chaim Sheba Medical Center, 52621 Tel Hashomer, Israel; ^2^Ozen Kashevet Hearing Clinic, 6 Ben Maimon Street, 92261 Jerusalem, Israel; ^3^Department of Communication Disorders, Hadassah Academic College, 37 Haneviim Street, P.O. Box 1114, 91010 Jerusalem, Israel; ^4^Speech & Hearing Center, Hebrew University School of Medicine, Hadassah Medical Center, Kiryat Hadassah, P.O. Box 12000, 91120 Jerusalem, Israel; ^5^Department of Medical Neurobiology (Physiology), Institute for Medical Research Israel-Canada, Hebrew University, Hadassah Medical School, P.O. Box 12272, 91120 Jerusalem, Israel

## Abstract

The mechanism of human hearing under water is debated. Some suggest it is by air conduction (AC), others by bone conduction (BC), and others by a combination of AC and BC. A clinical bone vibrator applied to soft tissue sites on the head, neck, and thorax also elicits hearing by a mechanism called soft tissue conduction (STC) or nonosseous BC. The present study was designed to test whether underwater hearing at low intensities is by AC or by osseous BC based on bone vibrations or by nonosseous BC (STC). Thresholds of normal hearing participants to bone vibrator stimulation with their forehead in air were recorded and again when forehead and bone vibrator were under water. A vibrometer detected vibrations of a dry human skull in all similar conditions (in air and under water) but not when water was the intermediary between the sound source and the skull forehead. Therefore, the intensities required to induce vibrations of the dry skull in water were significantly higher than the underwater hearing thresholds of the participants, under conditions when hearing by AC and osseous BC is not likely. The results support the hypothesis that hearing under water at low sound intensities may be attributed to nonosseous BC (STC).

## 1. Introduction

Even though the mammalian ear is adapted mainly for hearing in an air environment, that is, by air conduction (AC), involving the tympanic membrane and the middle ear ossicular chain, mammals including humans also hear under water [1–5]. However, the mechanism responsible for the hearing of sound in water is still not clear. Some studies support the tympanic theory which suggests that when under water, sound waves are conducted to the inner ear via the middle ear as in AC hearing [3]. That is, a passive traveling wave is induced, leading to activation of the outer hair cells [6]. Other studies provided evidence supporting an osseous bone conduction (BC) mechanism for underwater hearing, in which the sound field in the water surrounding the head induces skull bone vibrations that are necessary for eliciting BC hearing [1, 2, 5]. These bone vibrations lead to ossicular chain inertia, cochlear compression-distortion, cochlear fluid inertia, and radiation to the external canal, if occluded (occlusion effect) [7]. Some researchers have suggested a dual path theory of underwater hearing which assumes that both mechanisms, AC and BC, are involved in underwater hearing [2, 3].

Recently, further analysis of the mechanisms of BC has led to the suggestion that during low intensity BC stimulation hearing can result from transmission of sound waves to the inner ear via soft tissue and fluid pathways, for example, by delivering vibratory stimulation to sites on the head, neck, and thorax not overlying skull bone [8–10]. This mode of hearing has been referred to as soft tissue conduction (STC) [11] or nonosseous BC [12], and both terms will be used here interchangeably. The sound field in water is actually in initial, direct contact with the skin and other soft tissues (subcutaneous fat, muscle, etc.) overlying skull bone. Therefore, there can be a possible contribution of a nonosseous BC mechanism to underwater hearing at low sound levels. Since the established view of BC is based on the induction of actual vibrations of skull bone [7], the latter mode is called here osseous BC.

The present study was therefore designed to test the hypothesis that underwater hearing at low sound intensities can be elicited by a mechanism which does not involve osseous BC or AC (i.e., the external auditory meatus and middle ear) but rather by nonosseous BC. For this purpose, two complementary experiments were conducted, and their results are compared. The first experiment, on human subjects, was designed to assess the intensity levels required to elicit hearing* thresholds* under water (while AC hearing is compromised) and in air. The second experiment was designed to assess, with a vibrometer, the intensity levels required to induce detectable* skull vibrations* under water and in air. This second experiment was conducted on a dry human skull since it has been shown that the presence of soft tissues over and in the skull attenuates skull bone vibrations [13–16], so that a dry human skull represents a more sensitive model of the human head; if vibrations could not be detected on a dry skull, then they surely would not be found on an intact head with overlying, attenuating soft tissues.

## 2. Methods and Results

### 2.1. Experiment  1: Air and Underwater Hearing* Thresholds* of Normal Hearing Participants

#### 2.1.1. Participants

This experiment was conducted on six healthy participants (2 males; 4 females) with age ranging between 22 and 30 years (mean = 27.3, SD = 2.9). All participants had normal hearing AC and BC thresholds: 15 dB HL or better at frequencies 0.5 kHz, 1.0 kHz, 2.0 kHz, and 4.0 kHz. During all experiments, the participants were equipped with deeply inserted earplugs in both ears (Classic Superfit-30 Aero Co, E-A-R, USA) having a 30 dB noise reduction rating, as one of the ways used in this study to reduce the possibility that air conducted (AC) sound coming from the bone vibrator would excite the ear through the external auditory meatus and middle ear.

The entire experimental protocol was reviewed and approved by the Tel Aviv University Institutional Ethics Committee. All participants gave their informed written consent to take part in the study.

#### 2.1.2. Apparatus

A standard clinical B-71 (Radioear) bone vibrator was used as the sound source for assessing BC hearing thresholds to forehead stimulation in air and under water. A clinical audiometer (MAICO 41) generated stimuli at 0.5 kHz, 1.0 kHz, 2.0 kHz and 4.0 kHz delivered to the bone vibrator. Initially the BC thresholds were assessed with the forehead of the participants in air, with the bone vibrator pressed directly to the center of the forehead with an application force of 500 gram (approximately 5 N) provided by the P3333 Radioear headband, as in clinical audiometry. In the underwater stage of the experiment, the bone vibrator was tightly wrapped in a waterproof surgical rubber glove and then submerged in a water-filled round basin (diameter 48 cm, height 20 cm, with water to a depth of 14 cm). In order to ensure that the bone vibrator did not touch the walls of the basin, the bone vibrator was suspended by a string in a fixed position, at a constant distance from the forehead. BC* thresholds* when the forehead was in air and under water were expressed in dB HL settings of the audiometer in BC mode. However, the bone vibrator and audiometer in BC mode had been calibrated in accordance with ANSI specification (ANSI S3.6-2010) for delivering BC stimulation to the skin over the mastoid or forehead in the clinic and not for inducing a sound field when in contact with water. Therefore, the difference between the dB HL settings of the audiometer at* threshold* in air and under water of the participants was also calculated.

#### 2.1.3. Procedure

Hearing thresholds for all tested conditions (in air and in water) were obtained using an adaptive seeking threshold procedure following the 5 dB-up 10 dB-down rule similar to that used in clinical audiometry. The order of the tested frequencies was randomized between and within participants.

In order to determine underwater* thresholds* of the participants, the head was tilted so that only the forehead was immersed in water, and the clinical bone vibrator was submerged, not touching the forehead. The forehead was chosen for immersion because it is the most convenient site to submerge while at the same time the eyes, nose and the external ears (equipped with earplugs) of the tilted head were still above water. The nose in air also enabled the participants to breath normally. The center of the segment of the forehead under water was the same site to which bone vibrator had been pressed (with 5 N force) in air. The participants immersed their forehead by tilting their head into the basin of water, while the back of the head (inion) and the top of the head (parietal region) remained in air. As the forehead was gradually immersed in the water, the participants reported that the loudness of the sound gradually increased, reaching a maximum at a particular depth. Loudness sensation reached maximum when the forehead was about 4-5 cm under water, at which time the diameter of the segment of the immersed forehead was about 8 cm, and the forehead in water was 13 cm from the submerged bone vibrator. This latter condition was the one used for the remainder of this experiment. The hearing thresholds of the participants were also assessed at the same frequencies using an “in air control,” that is, with the forehead lifted from the water into air, 2 cm above the surface of the water, while the bone vibrator remained in water. This was done in order to further confirm that participants were not responding to air conducted sounds coming from the bone vibrator in water by AC hearing through the external auditory canals in air (even though they were equipped with earplugs).

In order to avoid order effects, testing conditions were randomized between participants. The tests were conducted at least twice for each condition to ensure repeatability.

**Figure 1:**
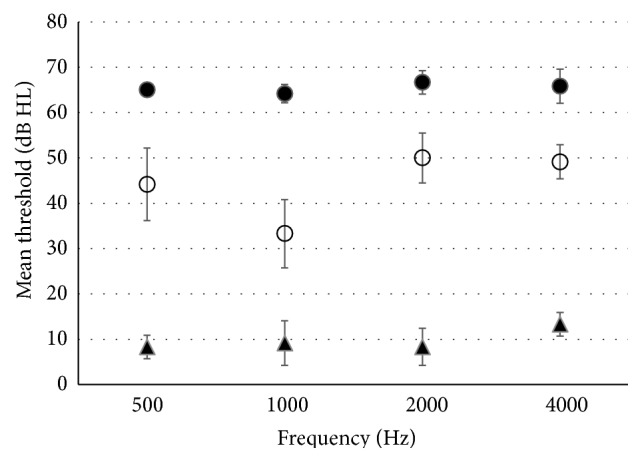
Mean (±SD) intensities eliciting threshold of normal hearing participants in the following conditions: [●] bone vibrator in water, forehead in air; [○] bone vibrator and forehead under water, not touching; [▲] bone vibrator directly on forehead, both in air.

*Results Experiment  1: Thresholds.* The mean (and standard deviation) thresholds of the same participants in air and under water are shown in Figure 1. It can be seen that better (i.e., lower) thresholds (mean 8–13 dB HL, depending on frequency) were obtained when the bone vibrator was directly applied to the forehead in air with a force of about 5 N. Higher thresholds (33–50 dB HL) were obtained under water, when water was the intervening medium between the bone vibrator and the forehead, 13 cm apart. The difference between these underwater-air thresholds ranged from 24 to 42 dB depending on frequency. Even higher (poorer) thresholds (60–70 dB HL) were obtained in the control procedure in which the head with earplugs was just above the water, while bone vibrator was still under water. A 2-way repeated measures ANOVA separating main effects of test condition and frequency confirmed these observations [*F*(1,71) = 737.927, *p* < 0.001, *F*(3,71) = 8.566, *p* = 0.001, resp.]. Pairwise multiple comparisons (Tukey test) found a significant difference between all tested conditions (*p* < 0.001). Also a significant interaction between frequency and test condition was found [*F*(6,71) = 7.605, *p* < 0.001]. The significant interaction resulted from better threshold at 1.0 kHz in the underwater condition (*p* < 0.001).

### 2.2. Experiment  2: Dry Skull* Vibrations* in Air and under Water

In order to assess the intensity levels of the stimuli which induce skull bone vibrations, the vibrations of a dry skull were measured using a Laser Doppler Vibrometer (LDV) in the two conditions similar to those in experiment  1 on the live participants, that is, when the bone vibrator and skull were both in water and when the bone vibrator was directly applied with 5 N force to the skull forehead in air.

#### 2.2.1. Apparatus

The vibration measurements were conducted on a dry adult skull using a LDV VibroMet model 500V single point vibrometer (MetroLaser, Inc., Irvine CA, USA) with a frequency range: DC to 70 kHz; velocity range: 2 *μ*m/s to 1 m/s. Recordings were conducted using the Metrolab software with a sampling rate of 40 kHz and frequency resolution of 10 Hz. To improve the signal-to-noise ratio, each measurement was averaged 100 times at each stimulation frequency, and the noise floor was carefully monitored during the entire procedure. In order to enhance laser beam reflection and reduce the noise floor, aluminum foil reflecting tape was pasted to all target areas, and the laser beam was focused on this reflector. A clinical audiometer (Interacoustics AC-33) was used in order to generate stimuli at 0.5 kHz, 1.0 kHz, 2.0 kHz, and 4.0 kHz delivered to a B-71 (radio ear) bone vibrator.

#### 2.2.2. Sensitivity Validation of the LDV System

The sensitivity of the LDV system was initially assessed by focusing the laser beam directly on a reflector applied to the center of the bone vibrator without any application force, that is, “unloaded.” Assessing the sensitivity of the vibrometer on the unloaded bone vibrator would provide its optimal sensitivity, since loading (pressing) the bone vibrator onto some surface (e.g., the forehead) would reduce the vibrations and lead to an elevation of the intensity required to elicit them. Vibrations of the bone vibrator were assessed for the same four frequencies in order to determine the lowest vibration velocity which could be detected by the LDV and the stimulus intensity required to induce it. The LDV detected vibrations at the same four frequencies directly on the bone vibrator with a magnitude ranging from 0.02 to 0.05 mm/sec at 0 dB HL. The noise floor in these measurements was 8 to 12 *μ*m/sec depending on frequency, and all were recorded with a signal-to-noise ratio (SNR) of at least 6 dB.

#### 2.2.3. Procedure

The* vibrations* of the dry skull were measured when it was entirely in air and the bone vibrator was applied to the forehead of the skull using the standard bone vibrator headband (direct application force equivalent to approximately 5 N), as during the hearing threshold determinations of the participants in experiment  1. The skull was placed on a spongy surface to avoid sound and vibration reflection from the table to the skull when delivering bone conduction stimuli to the skull. The LDV laser beam was focused at a reflector pasted on the back of the skull (inion-occipital protuberance) perpendicular to the surface of the skull, and pure-tones were delivered by the bone vibrator.

Skull bone vibrations under water were assessed under the same conditions as in experiment  1 in which human thresholds were determined (i.e., skull, suspended, tilted from above, skull forehead under water, inion in air, same water-protected bone vibrator under water at a distance of 13 cm from the tilted skull forehead under water, stimuli delivered by the bone vibrator under water, LDV laser beam focused to the inion of the skull in air, and LDV measuring skull vibrations at that site). In addition, vibrations were also assessed with the LDV beam focused on the parietal region of the skull, which was also still in air above the water, but much closer to the surface of the water than the assessment at the inion. The top of the parietal region of the skull is perpendicular to a line between the forehead and the inion. Furthermore, to confirm that the LDV could indeed measure skull vibrations induced under water, LDV recordings were conducted while the bone vibrator was manually pressed in direct underwater contact with the forehead of the immersed skull. In this latter condition, 13 cm of water no longer intervened between the sound source in the water and the skull forehead in water, and the LDV beam was directed to the inion. LDV recordings were conducted at least two times for each condition to confirm repeatability. Throughout all measurements in air and in water, vibrations were assessed when the stimulus intensities delivered began with the maximum output of the audiometer at each frequency, down to the lowest intensity at which bone vibrations could still be clearly detected by the LDV. Throughout all LDV measurements (directly on the bone vibrator and on the skull, both in air and in water), the noise floor was consistently between 8 to 12 *μ*m/sec, and the signal-to-noise ratio was at least 6 dB.

*Results Experiment  2: Skull Vibrations.* The magnitude of the vibrations of the skull (expressed as velocity, mm/sec) was measured by the LDV at the inion of the skull (*in air*) in response to stimulation by the bone vibrator pressed directly to the forehead (*in air*) with an application force of approximately 5 N (headband). The lowest stimulus intensities at which vibrations could be detected (6 dB above the noise floor) on the skull in* air* were 10 dB HL at 0.5 kHz, 30 dB HL at 1.0 kHz, 20 dB HL at 2.0 kHz, and 25 dB HL at 4.0 kHz. At lower intensities, the LDV could not detect any vibrations above the noise floor (which was 8 to 12 *μ*m/sec, depending on frequency). The magnitude of the vibrations increased linearly with stimulus intensity at each frequency, reaching highest levels at the maximal output of the audiometer.

When the forehead of the skull was suspended from above* in the water* basin, with the bone vibrator also in water 13 cm distant, bone vibrations above the noise floor were detected only at 1.0 kHz and then only at 65–70 dB HL (the maximum output of the audiometer at 1.0 kHz was 70 dB HL). At all other frequencies and intensities, vibrations could not be detected above the noise floor (8 to 12 *μ*m/sec), even at maximum output of the audiometer (which was 60 dB HL at 0.5 kHz, 70 dB HL at 2.0 kHz, and 80 dB HL at 4.0 kHz). Similar results (absence of vibrations with forehead under water at 0.5 kHz, 2.0 kHz, and 4.0 kHz, present at 1.0 kHz, but only at maximum intensities) were obtained when the LDV was focused at the top of the parietal region in air, above water. Bone vibrations were also determined when the bone vibrator in the water bath was manually pressed in direct contact with the forehead of the immersed tilted skull, without intervening water. In that situation, vibrations were clearly detected at the inion in air by the LDV with the same order of magnitude as those seen when the skull was in air with the bone vibrator applied to the forehead with the headband.

## 3. Discussion

The present study was designed to test the hypothesis that underwater hearing is elicited by a nonosseous BC (STC) mechanism, and not by AC, and not by osseous BC. This was achieved by determining the* thresholds* of normal participants in air and under water (experiment  1) and comparing them to the minimal intensities required to induce skull bone* vibrations* in air and under water (experiment  2).

### 3.1. Absence of AC Hearing under Water

Several aspects of the experimental design confirm that AC hearing was not involved: the ears were occluded by earplugs having a noise reduction rating (NRR) of 30 dB; the external ear canals of the participants were above water, while the sound source was under water. Since there is a large difference in the acoustic impedances of air and of water, the sound pressures induced by the bone vibrator in water would be largely reflected at its boundary with air and therefore not transmitted to the air above [17–19]. Furthermore, when the forehead of the participants was just above the surface of the water and the bone vibrator was still submerged in water (in air control), the thresholds were even higher compared to the condition in which the bone vibrator and forehead were both submerged in water, but not touching.

### 3.2. Absence of Osseous BC Hearing under Water

When the bone vibrator was pressed to the forehead of the skull directly with a 5 N force in* air*, the lowest intensities at which skull* vibrations* were clearly detected at the inion and at the parietal region were 10 to 30 dB HL (depending on frequency). On the other hand, the* thresholds* of the participants obtained when their foreheads and the bone vibrator were both* under water*, about 13 cm apart, were 24 to 42 dB (depending on frequency) higher than the thresholds obtained when the bone vibrator was pressed in air directly to the forehead of the participants with a 5 N force (experiment  1). Note that in the underwater condition, water served as the intermediary between the bone vibrator and the skull, and* vibrations* were detected at the inion only at 1.0 kHz and then only at the maximum output at that frequency (65 to 70 dB HL). No* vibrations* were detected at the other frequencies, even at the maximum intensities available. However, when the bone vibrator was pressed under water directly to the skull forehead (without water serving as the intermediary),* vibrations* were detected at the same frequencies and intensities as those obtained when the bone vibrator was applied to the forehead in air.

**Figure 2:**
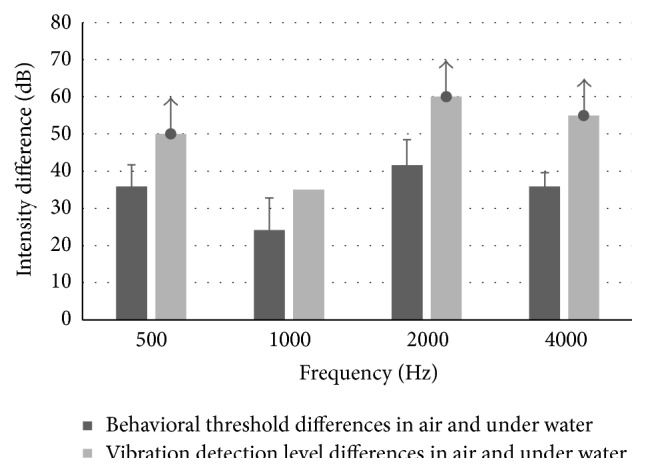
Bar graphs showing the air-underwater intensity differences in dB which elicited* threshold* in normal participants and which induced* vibrations* of skull bone at the 4 stimulus frequencies. Since the intensities delivered under water to the skull at 0.5, 2.0, and 4.0 kHz even at the maximum output of the audiometer did not induce skull vibrations, the maximal output was used in the calculation of the intensity differences for skull vibration, with an upward pointing arrow, to indicate that the intensity differences at these frequencies were even greater. For 1.0 kHz, the intensity used in this calculation is that which induced skull vibrations.

In both underwater parts of the experiments (*thresholds* of participants and skull* vibrations*), the test conditions for the determination of human* thresholds* and the assessment of dry skull* vibrations* were identical: the same water bath, the same bone vibrator wrapped with the same rubber glove, the immersion of the forehead in the same position, the bone vibrator in contact with water, and the same distance (13 cm) between the forehead and the submerged bone vibrator. Therefore, results of these two experiments can be compared, and it was seen that the* thresholds* of the participants under water were much lower than the dB HL settings required for induction of* vibrations* at the inion of the skull in air while the skull forehead was under water. However, the BC thresholds of the participants with their forehead* under water*, expressed in dB HL audiometer settings in BC mode of the audiometer, could not be directly compared to those when the bone vibrator was pressed (5 N) to the forehead in* air*. This is due to the audiometer and bone vibrator having been calibrated for BC stimulation when pressed to head sites (mastoid or forehead), and not for inducing a sound field in water. The mechanical impedances of the forehead and that of water are very different. However, this limitation can be overcome by comparing, in addition, the air-water* threshold* differences to the air-water intensity differences required to induce skull bone* vibrations*. If one assumes that only an osseous BC mechanism was involved in underwater hearing, then the* difference* between the intensity levels in air compared to that under water between experiments 1 (*thresholds*) and 2 (*vibrations*) would have been similar. In other words, if in both experiments only an osseous BC mechanism was involved in underwater hearing, then the same difference (between in air and under water; see Figure 2) would have been expected in experiments 1 and 2. However, as shown in Figure 2, a larger difference was found in experiment  2 (skull* vibrations*) than in experiment  1 (*threshold* of participants), suggesting the involvement of a different physiological mechanism in each of the two experiments. In fact, the exact difference with respect to the skull* vibrations* under water could not even be directly calculated for 0.5, 2.0, and 4.0 kHz, since no vibrations were detected at these frequencies on the inion of the skull when the skull forehead was under water, even with stimulation at the maximum output level of the audiometer. Vibrations on the skull under water were detected only at 1.0 kHz, at 65–70 dB HL. Therefore, in Figure 2, at the other frequencies at which skull vibrations were not detected, the maximum output of the audiometer was used for calculation of the intensity difference shown in the bar graph. An arrow pointing upward indicates that the intensity differences required for eliciting skull* vibrations* at these frequencies were even greater. As can be clearly seen in Figure 2, the intensity differences between those in air and in water required to induce skull bone* vibrations* were much greater than those needed to elicit* threshold* in the participants.

The possibility that the LDV system was not sensitive enough to detect vibrations of bone induced by the sound field in water at 0.5, 2.0, and 4.0 kHz is refuted by several findings. Among these, vibrations at 0 dB HL were detected by the LDV at these frequencies when focused directly on the bone vibrator under optimal conditions (bone vibrator unloaded) for sensitivity assessment of the vibrometer (see Methods, experiment  2, sensitivity). In addition, the LDV* was able* to detect skull vibrations at the inion in air at these frequencies, only when the stimuli were delivered by the bone vibrator applied in direct contact and pressed to the forehead in air using the headband. Skull vibrations were also detected by the LDV on the inion of the skull in* air* at these frequencies when the skull was tilted in* water* and the bone vibrator was pressed in direct contact under water to the forehead. In this situation, water was not serving as the intermediary between the sound field in water and the soft tissues over the bone at the forehead. Also, the noise floor during all LDV measurements was 10 to 11 *μ*m/sec, and a SNR of at least 6 dB was considered as the criterion for the presence of vibrations. On the other hand, the only situation at which the LDV did not detect vibrations of the skull at the inion and parietal region at 0.5, 2.0, and 4.0 kHz even at the maximum output in air at this SNR was when water served as the intervening medium between the bone vibrator and the skull forehead 13 cm apart. When water served as the intervening medium, vibrations were detected only at 1.0 kHz, but only at maximum output (65 and 70 dB HL). These findings lead to the suggestion that the sound field intensities induced by the bone vibrator in water at 0.5, 2.0, and 4.0 kHz (and at 1.0 kHz at intensities below 65–70 dB HL) were insufficient to induce skull bone vibrations. This, together with the fact that our participants were able to hear (*threshold*) sounds under water at intensities below those which induce skull bone* vibrations*, leads to the suggestion that other sound transmitting mechanisms (e.g., nonosseous BC) are likely involved at low sound intensities. Osseous BC may be involved in underwater hearing at higher stimulus intensities at sites on the head overlying bone (forehead, inion).

### 3.3. Nonosseous BC (STC) in Underwater Hearing at Low Intensities

The results of the present study therefore do not support the involvement of AC nor of osseous BC (at least not at low sound intensities) in underwater hearing. Since the sound field in water was actually in direct contact with the skin and the underlying additional soft tissues (muscle, adipose tissue, connective tissue, etc., of the forehead) overlying skull bone, it is suggested therefore that nonosseous BC can be considered an alternative mechanism for hearing in such conditions, without inducing skull vibrations, that is, without osseous BC [12]. In this mechanism, the sound field induced in the water can elicit vibrations in the soft tissues (STC) [11], since water and each of the soft tissues have similar acoustic impedances (defined as the product of the density of each tissue and the velocity of sound in that tissue; the acoustic impedance of water = 1.52 × 10^6^ kg/m^2^ sec and of typical soft tissues = 1.62 × 10^6^ kg/m^2^ sec). Therefore acoustic frequency vibrations can be transmitted from the water to the soft tissues of the body (similar to that during ultrasound diagnostic imaging [18]) to the inner ear fluids [17–19]. Thus, as a result of the* similarity* of acoustic impedances between water and the soft tissues of the head, the sound field pressure waves in water would be transmitted to the soft tissues and from there to the inner ear (i.e., low threshold stimulation by nonosseous BC-STC). Moreover, as the intensity of the sound field in water is gradually increased beginning at very low levels, for example, during threshold determinations, the nonosseous BC (STC) threshold would be reached at a lower intensity than the osseous BC threshold. On the other hand, when the acoustic impedances of adjacent tissues are very different from each other, for example, skin or soft tissue to bone (the acoustic impedance of bone = 7.8 × 10^6^ kg/m^2 ^sec), most of the vibratory energy (60–69%) would be reflected at the water-bone interface and not transmitted [17–19] and would not induce skull vibrations (osseous BC) at low intensities. Thus, due to the large differences in acoustic impedance between water and bone, the sound field in water and soft tissues at low intensities would be reflected at the bone and would not induce skull bone vibrations, so that osseous BC thresholds would be elevated. Thus, it is not likely that the sound field induced in water at* threshold* intensities would be able to induce vibrations of skull bone. This is similar to the considerations of the transmission of sound vibrations from an air environment in the external ear to the fluid environment in the inner ear, where the transmission loss is reduced by the impedance matching functions of the middle ear (tympanic membrane/stapes footplate area and lever ratios) [17, 19]. Furthermore, the presence of soft tissues overlying and within the skull actually attenuates the sound levels reaching the bone by about 20 dB [13–16] (such attenuation has also been shown when the magnitude of bone vibrations was evaluated with single point LDVs [15, 16]). This is likely a result of the differences in acoustic impedance between that of soft tissues and the underlying bone, so that a dry skull may be considered to provide a more sensitive measure of possible skull bone vibrations. If vibrations could not be detected on a dry skull, then they would surely not be detected on an intact head. The finding, however, that our participants (human head with soft tissues) reported* threshold* hearing at lower intensities than those required to induce skull vibrations supports the hypothesis that* vibrations* in the water and soft tissues which did not induce skull bone vibrations at most frequencies were nevertheless transmitted via nonosseous (STC) BC to the inner ear.

Additional support for a nonosseous BC (STC) mechanism can be derived from recent studies showing that hearing sensations can be elicited when the bone vibrator is applied to water or gel on the skin, without any real contact or application force, similar to the situation in underwater hearing [20]. Similarly, it has been suggested that a major part of the pathway for the transmission of water borne sounds to the inner ear of dolphins and other marine mammals, when they are totally under water, is through a soft tissue conduction pathway. In the dolphin, this pathway is a fat filled channel in the mandible [21].

This nonosseous-soft tissue mechanism for underwater hearing may also explain the hearing of the 20 weeks gestation fetus. The fetus is completely enveloped in amniotic fluid, with a softer skull and wider sutures between the skull bones, conditions in which actual skull vibrations (osseous BC) are not likely. Nonosseous BC (STC) therefore probably plays a significant role in hearing in a fluid environment at threshold levels.

## 4. Conclusion

It seems that underwater hearing at low sound intensities is mediated by a nonosseous BC (STC) mechanism. At higher intensities, there is likely a transition to osseous BC mechanisms which involves actual skull bone vibrations.
